# Myotoxicity of telbivudine in pre-existing muscle damage

**DOI:** 10.1186/1743-422X-7-323

**Published:** 2010-11-17

**Authors:** Josef Finsterer, Leyla Ay

**Affiliations:** 1 Department of Neurology, Krankenanstalt Rudolfstiftung, Vienna, Austria; 2 1st Medical Department, Krankenanstalt Rudolfstiftung, Vienna, Austria

## Abstract

**Objectives:**

It is unknown if telbivudine causes muscle damage only in patients with pre-existing muscle pathology.

**Case report:**

A 27 yo male of African origin received telbivudine for hepatitis B during 3 months. Three weeks after initiation of the drug he developed myalgia, and tiredness. Creatine-kinase increased from 278 U/l (n, <170 U/l) at baseline to 3243 U/l. Shortly after discontinuation of telbivudine muscle symptoms and hyper-CK-emia disappeared. The findings suggest that pre-existing muscle damage favored the myotoxic effect of telbivudine.

**Conclusions:**

Telbivudine appears to cause accelerated muscle toxicity if given to patients who already have muscle damage. Patients under telbivudine should be closely monitored for muscular side effects and those with pre-existing muscle damage should not receive the drug.

## Introduction

Telbivudine has been reported to cause moderate hyper-CK-emia in 9-12% of the treated patients [1,2]. The following case is important because it indicates that hyper-CK-emia from telbivudine occurs predominantly in patients with pre-existing subclinical muscle damage.

## Case report

The patient is a 27 yo HIV-negative male of African origin with a language barrier and a history of inactive hepatitis C, right-sided omalgia, a single episode of a polymorphic psychosis at age 26 y, mild chronic renal insufficiency (creatinine: 1.2 mg/dl (n, <1.1.mg/dl)) at least since age 25 y, mild recurrent hyper-CK-emia, leucopenia, and thrombopenia, recurrent abdominal pain presumably from chronic pancreatitis, and hyper-gammaglobulinemia (table 1). His family history was negative for neuromuscular disorder.

**Table 1:** Blood chemical values before, during (April to June 2009) and after telbivudine treatment in the described patient

Parameter	Reference value	1.6.07	1.6.07	241208	25.1208	19.3.09	8.4.09	3.7.09	6.7.09	7.7.09	9.7.09	10.7.09	21.7.09
Leucocytes	4.0-9.0/nl	4.6	5.4	5.2	5.1	**3**	**3.3**	**3.6**	**3.6**	**3.2**	nd	**2.7**	**3.3**
Thrombocytes	150-450/nl	**129**	153	**108**	**119**	**127**	**119**	**121**	**124**	**122**	nd	**112**	**119**
Creatine-kinase	-170 U/l	**212**	nd	159	**278**	nd	nd	**3243**	**2816**	**2034**	**2352**	**2202**	**1210**
GOT	-34 U/l	**144**	**377**	**40**	**40**	nd	nd	nd	**163**	**121**	nd	nd	**71**
GPT	-44 U/l	**118**	**375**	**57**	**60**	nd	nd	nd	**89**	**69**	nd	nd	**51**
GGT	-54 U/l	**147**	**278**	**63**	**69**	nd	nd	nd	48	40	nd	nd	36
Creatinine	-1.1 mg/dl	**1.2**	1.1	**1.43**	**1.13**	**1.5**	**1.4**	**1.23**	**1.13**	**1.2**	**1.3**	**1.3**	**1.13**
GFR	>90 ml/min/1.73	nd	nd	**64**	**83**	**60**	**65**	**75**	**83**	**77**	**70**	**70**	**83**
Alpha-amylase	28-100 U/l	**112**	97	**121**	**124**	nd	nd	**127**	**118**	**125**	nd	nd	**124**
Lipase	13-60 U/l	**77**	53	**72**	55	nd	nd	nd	nd	**80**	nd	nd	nd
Gamma-globulins	10-19%	nd	nd	nd	nd	**20.7**	**19.9**	nd	nd	**20.3**	nd	nd	nd

GOT: glutamate-oxalate transaminase, GPT: glutamate-pyruvate transaminase, GGT: gamma-glutamyl-transpeptidase, GFR: glomerular filtration rate, Nd: not done

At age 24 y hepatitis B was diagnosed. The patient received various antiviral therapies, such as lamivudine, adefovir dipivoxil, and fenofovir always for a short time because of low compliance, but effectively reducing the virus load as long as he agreed to take the drug. In April 2009 a therapy with telbivudine was started without performing a pre-treatment resistance test. Three weeks after initiation of telbivudine the patient experienced myalgias and tiredness. Although creatine-kinase (CK) had been elevated at least since age 25 y, it further increased 10-15 fold since initiation of telbivudine (table 1) why it was discontinued in June 2009. In addition to the muscle problems he developed leucopenia, which had been occasionally observed already previously (table 1). Glutamate-oxalate transaminase, glutamate-pyruvate transaminase, gamma-glutamyl transpeptidase, alpha-amylase, and lipase remained mildly elevated and the thrombocyte count and the glomerular filtration rate mildly declined before, during, and after telbivudine treatment (table 1). HBsAg, HBcAb, HBc-IgM-ab, and HBeAg were positive. Troponin-T was always normal and serum lactate at rest as well. Clinical neurologic examination eleven days after discontinuation of telbivudine revealed generally reduced tendon reflexes exclusively. Whether reduced tendon reflexes were due to neuropathy or myopathy remains speculative since the patient refused to undergo nerve conduction studies and electromyography.

## Discussion

Telbivudine is a L-nucleoside analogue used for the treatment of chronic hepatitis-B in adult patients with compensated hepatopathy and indication of ongoing viral replication [3]. The normal dosage is 600 mg/d and therapy should be continued at least until the HBeAg or HBV-DNA become negative and the anti-HBe becomes positive, until HBs seronegativity, or if the agent is ineffective. Like all other nucleoside analogues, telbivudine inhibits polymerase gamma (POLG1), which is responsible for mtDNA replication [1]. Depletion of mtDNA is associated with mitochondrial disease, including myopathy and lactacidosis [1].

Hyper-CK-emia and myalgia have been repeatedly reported in patients under telbivudine [4-6]. In a study on 105 patients treated with telbivudine the main adverse reactions were myalgia and general weakness [4]. In a study on 1370 patients with hepatitis B asymptomatic hyper-CK-emia was more common in patients receiving telbivudine than lamivudine [5]. According to the GLOBE-study telbivudine was associated with asymptomatic hyper-CK-emia more frequently than lamivudine (12.9 vs. 4.1%) [6]. In a Chocrane review (worldwide net of scientists and physicians, which aims at providing systematic reviews about the assessment of medical treatments) fatigue and malaise were found in 12-14% of the cases, asymptomatic hyper-CK-emia in 9%, and definite myopathy in 0.5% of the patients taking telbivudine [2].

In the instruction leaflet the manufacturer mentions that telbivudine-induced muscle disease may develop weeks or months after starting the therapy. Contrary to this statement the presented patient developed muscle symptoms and hyper-CK-emia already three weeks after initiation of the drug. The rapid development of the CK-increase may be due to the pre-existing muscle damage. Pre-existing mild hyper-CK-emia may be attributable either to nucleoside-analogue therapy prior to telbivudine or to subclinical primary myopathy. Arguments for a pre-existing primary myopathy are the generally reduced tendon reflexes, the elevated CK, GOT, and GPT, the chronic pancreatitis, the psychotic episode, and the renal insufficiency, although it cannot be excluded that these abnormalities were due to previous anti-viral therapy or other causes.

Whether a patient with pre-existing muscle pathology more frequently develops hyper-CK-emia or drug-induced myopathy from telbivudine remains speculative, but the presented case suggests such a pathomechanism. Arguments for aggravation of pre-existing hyper-CK-emia by telbivudine are that muscle symptoms and CK-elevation started shortly after initiation of the drug and that CK promptly and markedly declined after discontinuation of the drug. Since muscle symptoms and hyper-CK-emia resolved shortly after discontinuation of telbivudine no muscle biopsy was initiated and the complaints attributed to the therapy with telbivudine. In patients with a language barrier it should be guaranteed that the patient receiving drugs with potential side effects is regularly asked for such side effects in his language and monitored by appropriate laboratory investigations recognized by the treating physician.

## Conclusion

This case shows that telbivudine may accelerate muscle damage if there is pre-existing muscle damage. Patients under telbivudine should be closely monitored for muscular side effects and those with pre-existing muscle damage should not receive the drug.

## Competing interests

The authors hereby disclose any financial or personal relationship with other people or organizations that could have inappropriately influenced this work.

## Consent

Written informed consent was obtained from the patient for publication of this case report. A copy of the written consent is available for review by the Editor-in-Chief of this journal.

## Authors' contributions

LA carried out the clinical examination of the patient and participated in the drafting of the manuscript. JF participated in the sequence alignment, design, literature search, and coordination. All authors read and approved the final manuscript.
